# Knowledge and attitudes towards ambulatory treatment of tuberculоsis in Kazakhstan

**DOI:** 10.1186/s12913-020-05413-0

**Published:** 2020-06-22

**Authors:** Meruyert Darisheva, Melissa Tracy, Assel Terlikbayeva, Baurzhan Zhussupov, Neil Schluger, Tara McCrimmon

**Affiliations:** 1 Columbia University Global Health Research Center of Central Asia, Almaty, Kazakhstan; 2 School of Public Health, Department of Epidemiology and Biostatistics, University at Albany, Albany, NY USA; 3 Kazakh National Medical University, Almaty, Kazakhstan; 4 Department of Epidemiology, Columbia University Mailman School of Public Health, New York, NY USA; 5 Columbia University School of Social Work, 1255 Amsterdam Avenue, New York, NY USA

**Keywords:** Tuberculosis, TB treatment, Kazakhstan, TB attitude, TB knowledge

## Abstract

**Background:**

Ambulatory based treatment of tuberculosis has been recently introduced in Kazakhstan. We sought to assess the attitudes of the general population, TB patients and their household members towards ambulatory TB treatment and identify how knowledge of TB is associated with these attitudes.

**Methods:**

New pulmonary TB cases and their household and community controls were recruited from three regions of Kazakhstan in 2012–2014. 1083 participants completed audio computer-assisted self interviews to assess their knowledge of TB and attitudes towards ambulatory care. Mixed effects logistic regression models were used to identify factors associated with attitudes toward ambulatory TB treatment.

**Results:**

The proportion of people who considered ambulatory TB treatment as appropriate was very low (24.9%). Positive attitudes towards ambulatory TB treatment were significantly associated with region of residence, higher level of education, family support and experience with TB. The association between sufficient tuberculosis knowledge and favorable attitude toward ambulatory treatment was stronger among community controls compared to TB patients and their family members**.**

**Conclusions:**

This study provides insight into attitudes toward ambulatory TB treatment among different groups and the specific influence of TB knowledge on these attitudes. Our findings can inform the process of integration of new TB treatment strategies and the development of appropriate education and advocacy programs in the general population.

## Background

Kazakhstan is an upper middle-income country with a population of 18.7 million [1]. Currently, Kazakhstan has one of the highest Multidrug-Resistant Tuberculosis (MDR TB) burdens in the world [2], with an MDR TB incidence rate of 26 per 100,000, and a drug sensitive TB incidence rate as high as 68 per 100,000 in 2018 [3]. The TB epidemic is complicated by the emergence of multidrug-resistant strains of TB that continue to increase and are associated with the worst treatment outcomes [2].

Post-Soviet Kazakhstan inherited the highly vertical Soviet model of tuberculosis control, based on active case finding, individualized TB treatment, and high levels of hospitalization [4]. TB treatment practices, including hospitalizations for long periods with significant levels of interruption, treatment default, and failure to standardize treatment regimens have likely contributed to a high level of drug resistance to anti-tuberculosis medications [5].

To control a growing epidemic of TB, Kazakhstan introduced the National Tuberculosis Program (NTP) and World Health Organization’s Directly Observed Treatment, Short-Course (WHO’s DOTS) strategy in 1998. Through these efforts, TB incidence rates declined in the country; nevertheless TB still remains a major public health concern in Kazakhstan due to the rapid increase of drug-resistant strains. Inpatient models of TB treatment are expensive for health systems [6]. Ambulatory treatment, administered in outpatient settings, has been found effective in multiple countries [7, 8]. Since 2011, WHO has been recommending ambulatory treatment of both drug-susceptible and MDR-TB in outpatient settings [9]. Treatment at the ambulatory level is more cost-effective, reduces the risk of nosocomial transmission of drug resistant strains, and facilitates patients receiving comprehensive health services, including psychosocial care and support [10–12]. Furthermore, ambulatory treatment helps to shift treatment closer to places where patients live and allows patients to be more independent [13].

As Kazakhstan historically relied on a hospital-based model of TB treatment, the implementation of ambulatory TB care faced many structural challenges. WHO experts think that Kazakhstan still conducts excessive hospitalization for TB, with a system that discourages ambulatory care [11], and that reforms of Kazakhstan’s primary health care system are necessary to avoid unnecessary hospitalizations for TB [14]. Following WHO recommendations, Kazakhstan has been moving toward an ambulatory model of TB treatment and cutting the numbers of hospital beds. TB services have been intergrated into primary care and include daily provision of medications, hospital-replacement technologies (day care, home hospital, video-controlled therapy, mobile team for those who can not adhere to DOTS, adverse effects and comorbid conditions treatment, and psychosocial counseling for TB patients). TB diagnosis can be made by TB specialist at primary care and need to be confirmed with the Centralized Medical Advisory Commission [15, 16].

For this model to succeed, patients and the general public must be accepting of this approach. However, little is known about patient, family, and general population preferences for ambulatory TB care models. It was proved that better knowledge can lead to positive attitude and subsequently to good practices [17]. Insufficient knowledge about the disease might contribute to stigmatization and negative attitudes toward TB patients being treated in primary care among general population [18].

The objective of this study was to assess attitudes toward ambulatory TB treatment among TB patients, their household contacts and community dwellers. In particular, we aimed to examine the association between TB knowledge and attitudes toward ambulatory treatment. Our findings will provide information facilitating the adoption of the new TB treatment strategies and the development of appropriate education and advocacy programs in Kazakhstan.

## Methods

This study utilizes data from a case-control study among new pulmonary TB cases (index case), and both a matched household control and community control; additional results have previously been published [19–21]. We selected three regions in Kazakhstan (Almaty City, Almaty Oblast and Kostanay Oblast) that represented a range of TB burden as determined by the epidemiological surveillance from the National TB Center [21]. Within these regions we employed a cluster sampling approach, using SAS 9.2 to randomly select sub-regions, and calculated estimated recruitment numbers based on incidence data in these sub-regions. Within each region, index cases were identified by TB doctors as pulmonary TB cases recently diagnosed (within 90 days). The doctor was trained to introduce the study to all new cases of TB and to refer them to be prescreened by research staff. During the prescreening interview, research staff introduced the study to potential participants, administered consent and conducted a short 10-min prescreening survey to identify whether the case met eligibility criteria. Eligibility criteria included: (1) age 18 years and older; (2) permanent residence address for 3 months or more; (3) speaking Russian or Kazakh language fluently; and (4) absence of severe psychiatric condition that might impede their ability to provide informed consent. To be included in the study, all index cases were required to have an adult household member available to serve as a control. Both household and community controls were matched on the following criteria: age difference within 10 years and same household or geographic area to ensure similar environmental factors and socio-economic status. Controls were also required to meet the same primary eligibility criteria as the index cases described above.

The community control was sampled either from the same building as the index case using a Kish table (urban settings) or within a limited geographic area (rural settings) by choosing random direction (pen method) from the index case residence, and was thus matched to the index case on geographic location. More information on selection is provided in the Supplement. In total, 1083 participants were recruited into the study, including 387 cases, 342 household controls, 354 community controls.

The study received approvals from the Columbia University Institutional Review Board, Kazakhstan’s National Scientific Center of Physiopulmonology (KNSCP) and the Center of Life Sciences of Nazarbayev University (CLS of NU).

### Data collection

Data was collected through 60-min audio computer-assisted self-interview (ACASI). The study instrument was programmed and presented in Kazakh or Russian in Illume Survey Manager. Interviews were conducted in private rooms, with a research assistant available to assist participants if needed. Data were collected from September 2012 to March 2014.

Sociodemographic information was collected on all participants, including age, gender, employment status, educational level, marital status, ethnicity, living in an urban or rural setting, and current debt. These data were used as covariates in the analysis.

Attitudes toward ambulatory TB treatment were assessed with the question: “When a person first discovers that he or she has tuberculosis, how should that person be treated: hospitalized, treated at home, or hospitalized and then continue treatment at home?” Participants answering “TB patients should be only treated in hospitals” were classified as having negative attitudes to ambulatory treatment. Participants answering “TB patients should be treated in hospital and then continue treatment at home” or “TB patients should be treated at home” were considered to have a positive attitude to TB treatment provided in outpatient settings.

The aggregated variable “TB knowledge” was created through combination of responses for three TB-related questions (TB signs and symptoms, route of transmission and way of treatment). Sufficient level of TB knowledge was defined as (1) correctly selecting at least three TB symptoms from a multiple-choice list; (2) correctly selecting the airborne route of transmission from a multiple-choice list; and (3) correctly selecting the statement that tuberculosis can be completely cured with specific drugs and treatment regimens [22].

Perceived social support was measured as an ordinal variable ranging from 1 (very strongly disagree) to 7 (very strongly agree) by using the ‘Multidimensional Scale of Perceived Social Support’ (MSPSS), which has been previously used in Kazakhstan [23]. This measure includes three subscales pertaining to perceived support from family, friends, and one’s significant other, respectively, with 4 items for each subscale; a score of 6 or higher on a given subscale was considered a high level of support. The MSPSS has been shown to have good internal and test-retest reliability and good validity [24].

### Data analysis

Statistical analysis was performed using R version 3.3.2. Univariate statistics were examined to describe the study population, determine whether values for all measures of interest were plausible and consistent, and to assess the amount of missing data. For categorical variables, we examined frequencies, whereas for continuous variables (i.e., age), we calculated the mean and standard deviation and checked for outliers. We performed Chi-square tests to examine the associations between the outcome and independent variables.

We used the function ‘glmer’ in package ‘lme4’ to fit a generalized (binomial) linear mixed-effects model with random intercept for each triplet. This modeling procedure allows us to appropriately account for the matching of controls to cases, and serves as an alternative to conditional logistic regression in this situation where case status is not our outcome of interest. We included all variables with epidemiological and statistical significance in the full model and used backward selection to find the optimal model. Variables that were significantly associated with the outcome at *p* < 0.05, as well as our primary exposure of interest (level of TB knowledge), were retained in the final model. To examine potential effect measure modification of the association between TB knowledge and attitudes toward ambulatory treatment by participant group (i.e., cases, family controls, and community controls), we added interaction terms to the final model.

## Results

Sociodemographic characteristics of study participants are shown in Table 1.

**Table 1:** Socio-demographic and other characteristics of the study population (*N* = 1083)

	Total		TB cases		Household controls		Community controls	
	(*N* = 1083)		(*N* = 387)		(*N* = 342)		(*N* = 354)	
Characteristic	n	%	n	%	n	%	n	%
Gender								
Male	507	46.8	207	53.5	126	36.8	174	49.2
Female	576	53.2	180	46.5	216	63.2	180	50.8
Age, in years (Mean [SD])	39.0 [13.1]		35.5 [12.8]		40.9 [12.8]		41.1 [12.8]	
18–29	316	29.2	158	40.8	78	22.8	80	22.6
30–39	270	24.9	95	24.5	85	24.9	90	25.4
40–49	236	21.8	66	17.1	84	24.6	86	24.3
50–59	197	18.2	54	14.0	73	21.3	70	19.8
≥ 60	64	5.9	14	3.6	22	64.0	28	7.9
Ethnicity								
Kazakh	699	64.5	259	66.9	228	66.7	212	59.9
Russian	226	20.9	77	19.9	66	19.3	83	23.4
Others	158	14.6	51	13.2	48	14.0	59	16.7
Marital status								
Married	747	69.0	241	62.3	266	77.8	240	67.8
Single	336	31.0	146	37.7	76	22.2	114	32.2
Level of education^a^								
Primary and secondary school	99	9.1	34	8.8	35	10.2	30	8.5
High school	400	36.9	153	39.5	118	34.5	129	36.4
Vocational education	361	33.3	128	33.1	121	35.4	112	31.6
Higher education	223	20.6	72	18.6	68	19.9	83	23.4
Current employment status								
Employed	660	60.9	229	59.2	209	61.1	222	62.7
Unemployed	423	39.1	158	40.8	133	38.9	132	37.3
Currently in debt^b^								
No	683	63.1	251	64.9	198	57.9	234	66.1
Yes	400	36.9	136	35.1	144	42.1	120	33.9
Region								
Almaty city	133	12.3	56	14.5	33	9.6	44	12.4
Almaty oblast	524	48.4	185	47.8	170	49.7	169	47.7
Kostanay oblast	426	39.3	146	37.7	139	40.6	141	39.8
High level of family support^c^								
No	203	18.7	66	17.1	67	19.6	70	19.8
Yes	880	81.3	321	82.9	275	80.4	284	80.2
High level of friend support^d^								
No	342	31.6	129	33.3	114	33.3	99	28.0
Yes	741	68.4	258	66.7	228	66.7	255	72.0
High level of support from significant other^e^								
No	179	16.5	55	14.2	63	18.4	61	17.2
Yes	904	83.5	332	85.8	279	81.6	293	82.8
Tuberculosis knowledge								
Insufficient	473	43.7	125	32.3	163	47.7	185	52.3
Sufficient	610	56.3	262	67.7	179	52.3	169	47.7
Attitude toward ambulatory TB treatment^f^								
Negative	815	75.3	280	72.4	247	72.2	288	81.4
Positive	268	24.7	107	27.6	95	27.8	66	18.6

^a^ “Primary and secondary school” reflect schooling up to grade 9, whereas “high school” reflects grades 10–11

^b^ Assessed with the question: “Are you currently in debt?”

^c^ High level of support is indicated by a score of 6 or more on the Family Subscale of the Multidimensional Scale of Perceived Social Support

^d^ High level of support is indicated by a score of 6 or more on the Friends Subscale of the Multidimensional Scale of Perceived Social Support

^e^ High level of support is indicated by a score of 6 or more on the Significant Other Subscale of the Multidimensional Scale of Perceived Social Support

^f^ Positive attitude toward ambulatory TB treatment is indicated by responses of “TB patients should be treated in hospital and then continue treatment at home” or “TB patients should be treated at home” to a question about how a newly diagnosed person with TB should be treated

The proportion of study participants with sufficient TB knowledge was highest among cases (67.7%), followed by household controls (52.3%) and community controls (47.7%). Ninety two percent of respondents correctly knew that TB can be completely treated with specific drugs and regimen. Ninety seven percent of respondents was willing to take a family member at home for further treatment after he/she completed the hospital treatment.

Among TB cases, 20% of respondents reported having someone from their family diagnosed with TB (vs. 13.8%. among community control) and 18.9% of TB cases reported having someone with TB among their frequent contacts (friends, neighbors, relatives) (vs. 21.2% among community control).

The majority of respondents answered that TB patients should be only treated in hospitals. The proportions of respondents with positive attitudes toward out-of-hospital TB treatment (either alone or in combination with hospitalization) were significantly higher among TB patients (27.6%) and their household controls (27.8%). Only 18.6% of community controls agreed that TB patients can be initially hospitalized and then continue treatment at home or receive treatment at home from the first day.

Bivariate analyses showed an association between positive attitude toward ambulatory TB treatment and TB knowledge and other covariates (Table 2). A positive attitude toward ambulatory TB treatment was significantly associated with the region (with higher proportions of respondents in Almaty oblast indicating positive attitudes at 34.0% vs. 14.8 and 20.3% in Kostanay oblast and Almaty city, respectively; *p* < 0.001); perceived family support (with 36.0% of participants with low family support reporting positive attitudes towards ambulatory TB treatment, compared to 22.2% among those with high family support; *p* < 0.001); and level of TB knowledge (with 28.2% of those with sufficient TB knowledge reporting positive attitudes, compared to 20.3% of those with insufficient TB knowledge; *p* < 0.001). Relations between perceived support from significant others and positive attitudes towards ambulatory TB treatment were similar to those observed for perceived family support, whereby those with higher levels of support considered the in-hospital TB care model as more appropriate. Friends’ support was not significantly associated with attitude toward TB treatment, nor were demographic or socioeconomic characteristics, including gender, age, marital status, level of education, ethnicity and current employment status.

**Table 2:** Bivariate associations between positive attitude toward ambulatory TB treatment and participant characteristics (*N* = 1083)

	Positive attitude toward ambulatory TB treatment (full or combined with hospitalization)^a^											
	Total			TB cases			Household controls			Community controls		
	(*N* = 1083)			(*N* = 387)			(*N* = 342)			(*N* = 354)		
Characteristic	n	%	*p*-value	n	%	p-value	n	%	*p*-value	n	%	*p*-value
Gender												
Male	134	23.3	0.228	59	28.5	0.687	39	31.0	0.317	36	20.7	0.331
Female	134	26.4		48	26.7		56	25.9		30	16.7	
Age, in years (Mean [SD])												
18–29	77	24.4	0.911	45	28.5	0.897	18	23.1	0.719	14	17.5	0.969
30–39	69	25.6		25	26.3		25	29.4		19	21.1	
40–49	54	22.9		16	24.2		22	26.2		16	18.6	
50–59	50	25.4		16	29.6		22	30.1		12	17.1	
≥ 60	18	28.1		5	35.7		8	36.4		5	17.9	
Ethnicity												
Kazakh	178	25.5	0.761	69	26.6	0.818	68	29.8	0.472	41	19.3	0.906
Russian	53	23.5		23	29.9		15	22.7		15	18.1	
Others	37	23.4		15	29.4		12	25.0		10	16.9	
Marital status												
Married	180	24.1	0.460	65	27.0	0.702	76	28.6	0.540	39	16.3	0.093
Single	88	26.2		42	28.8		19	25.0		27	23.7	
Level of education^b^												
Primary and secondary school	22	22.2	0.121	7	20.6	0.131	8	22.9	0.726	7	23.3	0.509
High school	86	21.5		37	24.2		30	25.4		19	14.7	
Vocational education	104	28.8		45	35.2		37	30.6		22	19.6	
Higher education	56	25.1		18	25.0		20	29.4		18	21.7	
Current employment status												
Employed	159	24.1	0.533	60	26.2	0.443	60	28.7	0.630	39	17.6	0.500
Unemployed	109	25.8		47	29.7		35	26.3		27	20.5	
Currently in debt^c^												
No	179	26.2	0.145	39	28.7	0.739	30	20.8	0.014	20	16.7	0.494
Yes	89	22.3		68	27.1		65	32.8		46	19.7	
Region												
Almaty city	27	20.3	< 0.001	11	19.6	< 0.001	7	21.2	0.001	9	20.5	0.002
Almaty oblast	178	34.0		72	38.9		63	37.1		43	25.4	
Kostanay oblast	63	14.8		24	16.4		25	18.0		14	9.9	
High level of family support^d^												
No	73	36.0	< 0.001	26	39.4	0.019	27	40.3	0.011	20	28.6	0.017
Yes	195	22.2		81	25.2		68	24.7		46	16.2	
High level of friend support^e^												
No	92	26.9	0.264	33	25.6	0.520	37	32.5	0.172	22	22.2	0.281
Yes	176	23.8		74	28.7		58	25.4		44	17.3	
High level of support from significant other^f^												
No	62	34.6	0.001	21	38.2	0.059	23	36.5	0.087	18	29.5	0.017
Yes	206	22.8		86	25.9		72	25.8		48	16.4	
Tuberculosis knowledge												
Insufficient	96	20.3	0.003	32	25.6	0.534	40	24.5	0.202	24	13.0	0.004
Sufficient	172	28.2		75	28.6		55	30.7		42	24.9	

^a^ Positive attitude toward ambulatory TB treatment is indicated by responses of “TB patients should be treated in hospital and then continue treatment at home” or “TB patients should be treated at home” to a question about how a newly diagnosed person with TB should be treated

^b^ “Primary and secondary school” reflect schooling up to grade 9, whereas “high school” reflects grades 10–11

^c^ Assessed with the question: “Are you currently in debt?”

^d^ High level of support is indicated by a score of 6 or more on the Family Subscale of the Multidimensional Scale of Perceived Social Support

^e^ High level of support is indicated by a score of 6 or more on the Friends Subscale of the Multidimensional Scale of Perceived Social Support

^f^ High level of support is indicated by a score of 6 or more on the Significant Other Subscale of the Multidimensional Scale of Perceived Social Support

Bivariate analyses stratified by the case-control status of respondents demonstrated that sufficient TB knowledge and positive TB attitudes were significantly associated among community controls (OR = 2.22, 95% CI = 1.28–3.86), but not among TB cases (OR = 1.17; 95% CI = 0.72–1.89) or household controls (OR = 1.36; 95% CI = 0.85–2.20).

The final mixed effect logistic regression model (Table 3) includes the following independent variables: case-control status, TB knowledge, level of education, region and perceived family support.

**Table 3:** Results of mixed effects logistic regression models predicting positive attitude toward ambulatory TB treatment

	Positive attitude toward ambulatory TB treatment			
Characteristic	beta	OR	95% CI for OR	*p*-value
Participant group				
Community controls	0.00	1.00	(ref)	–
Household controls	0.65	1.92	(1.25–2.95)	0.003
New pulmonary TB cases	0.73	2.07	(1.35–3.17)	0.001
Tuberculosis knowledge				
Insufficient	0.00	1.00	(ref)	–
Sufficient	0.14	1.15	(0.76–1.73)	0.501
Level of education				
Primary and secondary school	−0.55	0.58	(0.27–1.23)	0.156
High school	−0.68	0.51	(0.30–0.87)	0.013
Vocational education	0.04	1.04	(0.63–1.73)	0.866
Higher education	0.00	1.00	(ref)	–
Region				
Almaty city	0.37	1.45	(0.69–3.05)	0.328
Almaty oblast	1.51	4.55	(2.68–7.72)	< 0.001
Kostanay oblast	0.00	1.00	(ref)	–
High level of family support				
No	0.00	1.00	(ref)	–
Yes	−1.05	0.35	(0.22–0.55)	< 0.001

The multivariable analysis showed that living in Almaty oblast was associated with higher odds of positive attitude toward ambulatory TB treatment (OR = 4.55; 95% CI = 2.68–7.72 compared to living in Kostanay oblast), whereas reporting high family support was associated with lower odds of positive TB attitude (OR = 0.35; 95% CI = 0.22–0.55), adjusting for case-control status, TB knowledge, and level of education. Current TB experience in the form of being a TB patient or sharing a household with a TB patient was associated with a positive attitude toward ambulatory model of TB treatment (OR = 2.07; 95% CI = 1.35–3.17 for TB cases and OR = 1.92; 95% CI = 1.25–2.95 for household controls, both compared to community controls). In this adjusted model, sufficient level of TB knowledge was not significantly associated with positive TB treatment attitude (OR = 1.15; 95%CI = 0.76–1.73). High school education was associated with lower odds of positive TB attitude as compared to higher education (OR = 0.51; 95% CI = 0.30–0.87).

Given the evidence for effect modification of the association between TB knowledge and TB treatment attitudes by participant group in bivariate analyses, we added interaction terms for TB knowledge and respondent group in the full multivariable model (Table 4).

**Table 4:** Results of mixed effects logistic regression models predicting positive attitude toward ambulatory TB treatment, including interaction between level of TB knowledge and participant group (TB case, household control, community control)

Characteristic	Positive attitude toward ambulatory TB treatment			
	beta	OR	95% CI for OR	*p*-value
MAIN EFFECTS				
Participant group				
Community controls	0.00	1.00	(ref)	–
Household controls	1.03	2.79	(1.42–5.48)	0.003
New pulmonary TB cases	1.09	2.96	(1.44–6.08)	0.003
Tuberculosis knowledge				
Insufficient	0.00	1.00	(ref)	–
Sufficient	0.58	1.79	(0.89–3.61)	0.102
Level of education				
Primary and secondary school	−0.61	0.54	(0.25–1.15)	0.112
High school	−0.73	0.48	(0.28–0.82)	0.007
Vocational education	−0.02	0.98	(0.59–1.62)	0.926
Higher education	0.00	1.00	(ref)	–
Region				
Almaty city	0.44	1.55	(0.74–3.25)	0.248
Almaty oblast	1.52	4.58	(2.70–7.74)	< 0.001
Kostanay oblast	0.00	1.00	(ref)	–
High level of family support				
No	0.00	1.00	(ref)	–
Yes	1.04	0.35	(0.22–0.56)	< 0.001
INTERACTIONS				
Sufficient knowledge × household controls	−0.63	0.53	(0.21–1.34)	0.180
Sufficient knowledge × new pulmonary TB cases	−0.70	0.49	(0.20–1.23)	0.130

**Fig. 1:**
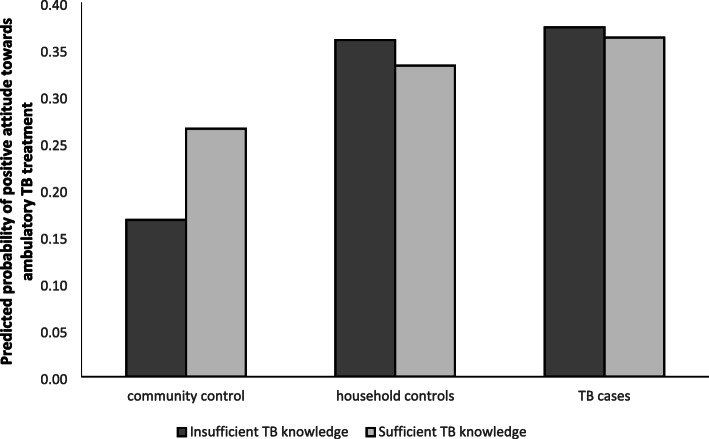
Predicted probabilities of positive attitude towards ambulatory TB treatment from the model with interactions*

Though the interaction between TB knowledge and case-control status was not statistically significant in this adjusted model (*p* = 0.235), the estimated interaction effects suggest that the association between attitude toward ambulatory TB treatment and TB knowledge varies dependent on disease status and on experience of living with someone who has had active TB. In particular, in the interactions model, level of TB knowledge has a marginally significant association with positive attitude towards ambulatory TB treatment among community controls (OR = 1.79; 95% CI = 0.89–3.61). Among those with sufficient TB knowledge, the association between current TB experience and the outcome variable is stronger for both TB cases (OR = 2.96; 95% CI = 1.44–6.08) and household controls (OR = 2.79; 95% CI = 1.42–5.48) as compared to community controls. These interaction effects are illustrated in Fig. 1.

## Discussion

The majority of respondents in all case-control groups strongly support the idea that TB should be only treated in hospitals. Although most of respondents in our study had “sufficient” TB knowledge, a significant proportion of respondents were not informed enough about TB (43.7%). Even though the proportion of community controls with sufficient TB knowledge was lower than the corresponding proportions among TB cases and household controls, TB knowledge among community controls was marginally associated with considering the ambulatory model of TB treatment as appropriate. Insufficient knowledge about the disease might contribute to stigmatization and fear toward patients [25]. The majority of respondents in the Report of a Joint IUAT/WHO Study Group said “they would not live with a TB patient” (93.8%), “would not share food, clothes or a bath with them” (95.4%), and “would not hug, kiss or touch them” (97%) [26]. Since TB is a communicable and high-burden disease, it is very important to develop positive attitudes toward TB patients and ambulatory TB treatment among the population of Kazakhstan. Findings that higher levels of knowledge about TB signs and symptoms, spread, and treatment were associated with more positive attitudes toward ambulatory TB treatment highlight the importance of continued education efforts.

Both TB patients and their family members had a more positive attitude toward ambulatory TB treatment than community controls, even in the face of insufficient knowledge about TB signs/symptoms, spread, and treatment. Ambulatory TB care gives TB patients more independence and helps to move treatment closer to places where patients live. In addition, administering TB treatment at outpatient settings might help to reduce the risk of nosocomial infection by resistant strains in hospitals [27]. Therefore, it is understandable that TB patients and their family members would view ambulatory TB treatment more favorably, although positive attitudes among these groups were still not nearly universal. TB treatment outcome and adherence are associated with level of social support including support from family members, partners, and friends [28, 29]. We considered that people who had social support were more likely to continue treatment at home. However, we found that respondents with higher levels of support from family members and significant others were less likely to view ambulatory TB treatment favorably. These results may be explained by fear of transmitting the disease to family members or significant others at home. These findings have substantial practical implications for the development of ambulatory TB services. In particular, it is crucial to communicate that the appropriate control measures can minimize the risk of TB transmission to other people.

Another potential barrier is availability and access to TB medical services in rural areas where 43% of the population live [1]. We found that the number of TB patients supporting the ambulatory model of care was higher in Almaty oblast rural areas. Urban populations have better access to services and health care facilities (hospitals and policlinics), while the rural areas are characterized by a lack of all facilities and less developed infrastructure.

Comorbidities among TB patients [30], alhocol abuse, social determinants of therapeutic failure such as low income and low education [31] should be considered for the effective outpatient treatment.

To promote ambulatory TB care, the population should be informed about new treatment strategies which allow patients to rapidly become non-infectious. TB treatment outcomes in out-patient settings should be communicated to the general public to show that an ambulatory model of TB care produces at least the same results as in-hospital treatment. Such programs would reduce stigma against TB patients and provide necessary support from the community. Involvement of national leadership, mass media to reach target populations, and involvement of the local community are all required to create a truly effective campaign that takes into account cultural context and existing barriers. Educational programs on the route of TB transmission, signs and symptoms, consequences, prevention, types of treatment, and appropriate health messages are required to alter the negative social norms for a healthier society [32].

Multiple factors associated with TB treatment outcomes, organizational and structural barriers for TB services delivery must be addressed to successfully implement ambulatory TB treatment across Kazakhstan.

## Limitations

Our study population of index-cases was limited to those diagnosed with TB and registered with the local TB dispensary and met our inclusion criteria, thus excluding all incident TB- patients with no family control. The study was conducted in three regions, which limits the generalizability of findings. Not all possible confounders may have been measured and included in the analysis.

## Conclusions

More information and education campaigns about benefits of the new treatment strategy are needed to alter public opinion and attitude. Empowering communities through proper education would help to increase their TB knowledge and develop more positive attitudes toward ambulatory care of tuberculosis. Our findings can help to adapt and implement new treatment strategies in Kazakhstan and other Central Asia countries.

## Supplementary information

**Additional file 1.** Recruitment.

## Data Availability

The data that support the findings of this study are available from CLS NU but restrictions apply to the availability of these data, which were used under license for the current study, and so are not publicly available. Data are however available from the authors upon reasonable request and with permission of CLS NU. Data requests may be sent to corresponding author at meruyert.darisheva@ghrcca.org

## Abbreviations

MDR TB: Multidrug-resistant tuberculosis
NTP: National tuberculosis program
WHO: World health organization
DOTS: Directly observed treatment, short-course
KNSCP: Kazakhstan’s national scientific center of physiopulmonology
CLS of NU: Center of life sciences of Nazarbayev university
ACASI: Audio computer-assisted self-interview
MSPSS: Multidimensional scale of perceived social support
GHRCCA: Global health research center of central Asia
