# Supplementary material for: Knowledge and attitudes towards ambulatory treatment of tuberculоsis in Kazakhstan
Source: BMC Health Serv Res. 2020 Jun 22;20:563. doi: 10.1186/s12913-020-05413-0 (PMC7310255; doi:10.1186/s12913-020-05413-0)
Supplement: Supplementary file 1 — Additional file 1. Recruitment. [file 12913_2020_5413_MOESM1_ESM.docx]

*Study design and recruitment process*

Matched case-control design was used for data collection. At first stage we identified index cases which is newly detected pulmonary TB. Then a set of matching (age difference within 10 years, same household/geographic area assuming similar environmental factors and socio-economic status) were selected.

**Index-cases** were identified as pulmonary TB cases diagnosed within three months prior to data collection, corresponding to all incident TB cases registered between April 2012 and March 2014 in the selected districts.

The index cases of pulmonary TB were identified by having recently (in the prior three months) had either: (1) both a positive culture/positive smear examination and confirmation by nucleic acid amplification testing; or (2) clinical and radiographic results confirming tuberculosis and response to anti-tuberculosis treatment. This included all cases identified in the three months prior to study start, as well as all cases going forward until the study end.

**The household control** was matched with the index case by age (within ten years of the index case). Eligible household control participant should NOT have previously diagnosed case of pulmonary tuberculosis that adheres to the study case definition, based on self-reported information from participant at baseline and meet the universal eligibility criteria.

During after the prescreening process a list of number of eligible household contacts was developed per index case. If more than one eligible household contact is available, the Kish method was used to randomly select the household contact study participant.

During the prescreening of the index case information on existence of a potentially eligible household contact was collected. GHRCCA research staff visited the address of the index case, introduce the study to an adult who responds to the door and request to prescreen all adults in the home (the index case could potentially be included in this group if they choose not to disclose their participation in the study to those residing in the same household as them). Each adult member of the household was then be consented and prescreened in the household in an isolated space where there conversation cannot be seen or overhear. Upon confirmation that at least one household control exists and is potentially eligible for inclusion into the study, the research staff scheduled the screening and baseline interview date. If more than one member of the household is found to be eligible the Kish method was used to identify the potential study participant. Only numbers, not names of individuals was used in the Kish method at this time. The subject always was provided with a choice to have interview either at the clinic, at the research office, or at their home.

**Eligible community control** participant should NOT have previously diagnosed case of pulmonary tuberculosis that adheres to the study case definition, with confirmation from the local tuberculosis inpatient clinic at baseline meet the universal eligibility criteria: Be 18 years or older of age at study screening;Have a permanent address and have been residing at this current address for more than three months;Have other adult household members;

Speak Russian or Kazakh fluently; Does not have severe psychiatric or mental condition that could impede ability to provide informed consent and complete all study materials as assessed by research assistants during prescreening and screening.

Upon identification of an eligible case and household contact a community control was selected from the population. One community control was sampled from either the same building (urban setting) as the index case or within a limited geographic radius (rural) if the index case lives in a standalone structure. For urban settings all the residences within the building was enumerated based on visible mailboxes and given a number. Using a Kish table an apartment was randomly selected from the list for inclusion into the study. For rural settings the community control household is selected by choosing a random direction (pen method) from the index case's residence and selecting the 3rd dwelling among those along the line from the index's household to the edge of the community.

One community control participant was randomly selected from the same neighborhood as each index case. The selected household was visited by the study recruiters who introduced the study to adult household members and recruit them to the study. The study Recruiters asked of an adult in the household to number all adults in the household older than 18 years. If an eligible individual is available, the recruiters prescreen that individual. After prescreening, if more than one person is eligible, the recruiters randomly select only one potential community control from all prescreened persons using a Kish table. In case of nonresponse, the interviewers proceed to the next household. If an eligible and interested in participating individual is identified, the community control consented, enrolled to the study, and administered the baseline questionnaire. As with all participants, interviews may be conducted in the home, at the TB clinic, or at the GHRCCA research center, as selected by the participant.
